# Academic procrastination data of students in Makassar, Indonesia

**DOI:** 10.1016/j.dib.2020.106608

**Published:** 2020-11-29

**Authors:** Muhammad Rusdi, Nur Hidayah, Hetti Rahmawati, Imanuel Hitipeuw

**Affiliations:** a Faculty of Psychological Education Universitas Negeri Malang Indonesia; b STKIP Muhammadiyah Bone, South Sulawesi; c Faculty of Science Education, Universitas Negeri Malang Indonesia

**Keywords:** Academic procrastination, Fear of Failure and Task Aversiveness

## Abstract

This is a non-experimental study which aims to obtain the academic procrastination data of students at Makassar, South Sulawesi, Indonesia. It used the non-random sampling method to obtain data from 586 students, consisting of 310 men and 276 women through questionnaires. The data were then descriptively analyzed using an academic procrastination scale, which was compiled and developed based on the Solomon & Rothblum (1984) theory. This scale consist of two sections which aim to determine the areas or fields of academic function, and the reasons for academic procrastination.

## Specifications Table

**Table:** 

Subject	Educational psychology
Specific subject area	Vocational Psychology, Academic Procrastination.
Type of data	Table and Figure
How data were acquired	This data was obtained using an academic procrastination scale for students in Makassar.
Data Format	Raw data analyzed.
Parameters for data collection	Academic procrastination scale compiled and developed based on the Solomon & Rotbhlum (1984) theory.
Description of data collection	This data was obtained from an assessment that used an academic procrastination scale on 586 students in the XIII semester at Makassar, Indonesia. Students responded to statements by using a Likert-scale of 5,4,3,2,1, which represents strongly agree, agree, doubt, disagree, and strongly disagree, respectively.
Data source location	Universities located at Makassar in South Sulawesi, country: Indonesia. Makassar geographically is at 08′10′00-08′22′00′ South Latitude and 114,4-112′54′ East Longitude, and with a google map https://www.google.com/maps/@-5.1518379,119.4296153,16.5z
Data accessibility	The data available in Mendeley data: http://dx.doi.org/10.17632/8z9532ps65.10

## Value of the Data

• This data can be used to identify academic procrastination behavior in students, through the context of cultural differences.
• The data can also be used to investigate the influence of academic procrastination on the development of information technology.
• It provides new information to universities on how to improve the quality of learning in classrooms.
• This data can be used by lecturers to improve the quality of teaching service, thereby reducing academic procrastination behavior.

## 1 Data Description

Data was collected using questionnaires from 586 students consisting of 310 men (53%) and 276 women (47%). Structural equation modelling and factor analysis were used to validate the construct. Furthermore, it was descriptively analyzed using an academic procrastination scale, which was compiled and developed based on the Solomon & Rothblum (1984) theory [1,2]. In addition, the Procrastination Assessment Scale Students (PASS) further divides academic procrastination into two sections. The first section included 18 items focusing on areas of academic procrastination, such as (a) writing a term paper, (b) studying for an exam, (c) keeping up with weekly reading assignment, (d) performing an administrative task, (e) attending meetings, and (f) performing academic task in general. These items can be found in Table 1. The second section of academic procrastination consists of 26 items, measuring two factors: Fear of Failure and Task Aversiveness. The items of the first factor, Fear of Failure, focus on anxiety about meeting one's own standards and other people's expectations, fear of success and lack of self-confidence. The items of the second factor, Task Aversiveness, focus on the unpleasantness of the task, risk taking and lack of assertion. These items can be found in Table 2.

**Table 1:** Areas of Academic Procrastination.

No.	Items	Mean	SD	Loading Factor	Crombach Alpha	CV	AVE
**A1**	To what degree do you procrastinate on this task?	3,57	1,258	0,666	**0,949**	**0,950**	**0,512**
**A2**	To what degree is procrastination on this task a problem for you?	3,55	1,266	0,719			
**A3**	To what extent do you want to decrease your tendency to procrastinate on this task?	3,35	1,315	0,621			
**A4**	To what degree do you procrastinate on this task?	3,56	1,275	0,700			
**A5**	To what degree is procrastination on this task a problem for you?	3,66	1,193	0,743			
**A6**	To what extent do you want to decrease your tendency to procrastinate on this task?	3,69	1,283	0,782			
**A7**	To what degree do you procrastinate on this task?	3,46	1,151	0,727			
**A8**	To what degree is procrastination on this task a problem for you?	3,56	1,319	0,657			
**A9**	To what extent do you want to decrease your tendency to procrastinate on this task?	3,62	1,303	0,769			
**A10**	To what degree do you procrastinate on this task?	3,71	1,186	0,745			
**A11**	To what degree is procrastination on this task a problem for you?	3,48	1,263	0,721			
**A12**	To what extent do you want to decrease your tendency to procrastinate on this task?	3,58	1,202	0,727			
**A13**	To what degree do you procrastinate on this task?	3,44	1,338	0,687			
**A14**	To what degree is procrastination on this task a problem for you?	3,58	1,334	0,735			
**A15**	To what extent do you want to decrease your tendency to procrastinate on this task?	3,55	1,200	0,667			
**A16**	To what degree do you procrastinate on this task?	3,54	1,317	0,740			
**A17**	To what degree is procrastination on this task a problem for you?	3,67	1,231	0,701			
**A18**	To what extent do you want to decrease your tendency to procrastinate on this task?	3,58	1,198	0,743

**Table 2:** Reasons for Academic Procrastination.

No.	Items	Mean	SD	Loading Factor	Crombach Alpha	CV	AVE
**R19**	You were concerned the profession wouldn't like your work.	3,56	1,232	0,763	**0,97**	**0,970**	**0,554**
**R20**	You waited until a classmate did his or hers so that he/she could give you some advice	3,507	1,112	0,782			
**R21**	you had a hard time knowing what to include and what not to include in your paper.	3,37	1,189	0,756			
**R22**	You had too many other things to do.	3,55	1,181	0,736			
**R23**	There's some information you needed to ask the professor, but you felt uncomfortable approaching him/her.	3,509	1,287	0,74			
**R24**	you were worried you would get a bad grade.	3,35	1,129	0,711			
**R25**	You resented having to do things assigned by others.	3,62	1,102	0,79			
**R26**	You didn't think you knew enough to write the paper.	3,55	1,145	0,773			
**R27**	You disliked writing term papers.	3,44	1,137	0,739			
**R28**	You felt overwhelmed by the task.	3,43	1,269	0,71			
**R29**	You had difficulty requesting information from other people.	3,403	1,208	0,703			
**R30**	You looked forward to the excitement of doing this task at the last minute.	3,56	1,340	0,711			
**R31**	You couldn't choose between all the topics.	3,65	1,163	0,759			
**R32**	You were concerned that if you did well, your classmates would resent you.	3,43	1,169	0,746			
**R33**	You didn't trust yourself to do a good job.	3,69	1,218	0,746			
**R34**	You didn't have enough energy to begin the task	3,601	1,187	0,769			
**R35**	You felt it just takes too long to write a term paper.	3,41	1,193	0,741			
**R36**	You liked the challenge of waiting until the deadline	3,58	1,295	0,752			
**R37**	You knew that your classmates hadn't started the paper either.	3,56	1,211	0,741			
**R38**	you resented people setting deadlines for you.	3,45	1,164	0,724			
**R39**	You were concerned you wouldn't meet your expectations	3,43	1,121	0,711			
**R40**	You were concerned that if you got a good grade, people would have higher expectations of you in the future.	3,52	1,279	0,724			
**R41**	You waited to see if the professor would give you some more information about the paper.	3,65	1,155	0,777			
**R42**	You set very high standards for yourself, and you worried that you wouldn't be able to meet those standards	3,48	1,097	0,780			
**R43**	You just felt too lazy to write a term paper	3,63	1,313	0,76			
**R44**	Your friends were pressuring you to do other things	3,46	1,205	0,666			

## 2 Experimental Design, Materials, and Methods

Data was obtained from participants through a non-experimental method, using questionnaires, after which it was then descriptively analyzed [3]. This descriptive data was used to determine the level of students’ academic procrastination behavior.

Table 1 shows that the academic task areas and loading factors of each item are worth more than 0.50, with Crombach Alpha 0.949, CV 0.950, and AVE 0.512. From this result, it can be concluded that there is evidence of academic task areas having acceptable unidimensionality.

Table 2 shows that the reason for delaying academic assignment is worth more than 0.50, with Crombach Alpha 0.970, CV 0.970, and AVE 0.554. Therefore, it can be concluded that the reasons for procrastinating academic assignments have acceptable unidimensionality.

The participants of this study were students in the XIII semester at several universities in Makassar, South Sulawesi, Indonesia, during the 2020 academic year. It made use of the non-random sampling method to select participants from three universities, namely Makassar State University, Hasanuddin University, and the Department of Psychology, Makassar State Islamic University. Subsequently, questionnaires were distributed directly to 586 students at XIII semesters in Makassar, comprising of 310 men and 276 women. The data collection process took place from January 5 to 28, 2020.

An academic procrastination scale contains a total of 44 items, divided into two sections. The first section was used to determine the areas of academic procrastination. Scales for these areas have different labels, but in each case they were coded from 1 (strongly disagree) to 5 (strongly agree). The second section examined the reasons for procrastinating academic assignments, and it used a Likert scale coded from 5 (strongly agree) to 1 (strongly disagree), [4]. The associated data repository contains files for the original questionnaire, the location of the study, raw participant data, Construct Validity (CV) values, Average Variance Extracted (AVE) values, factor loadings (including means, standard deviations, and reliabilities), and analyses in the form of tables and figures.

## Ethics Statement

This research was reviewed and agreed upon by the institutional review board of the university in Makassar based on decree number 5.11.1/UN32.8.4.1/DT2019. Explanation and approval were obtained from each participant before data collection.

## Declaration of Competing Interest

This research was not sponsored by any institution or organization.

## Appendix Supplementary materials

Image, application 1

Image, application 2
